# Integrating child rights standards in contraceptive and abortion care for minors in Africa

**DOI:** 10.1002/ijgo.14502

**Published:** 2022-10-17

**Authors:** Godfrey Dalitso Kangaude, Catriona Macleod, Ernestina Coast, Tamara Fetters

**Affiliations:** 1 Critical Studies in Sexualities and Reproduction Program, Rhodes University, Makhanda, South Africa; 2 Department of International Development, London School of Economics & Political Science, London, UK; 3 Ipas, Chapel Hill, North Carolina, USA

**Keywords:** African charter on the rights and welfare of the child, child rights, contraceptive and abortion care, convention on the rights of the child, evolving capacities, WHO abortion care guideline

## Abstract

Minor girls in Africa face challenges in accessing high-quality contraceptive and abortion services because laws and policies are not child-friendly. Many countries maintain restrictive laws, policies, or hospital practices that make it difficult for minors to access contraception and safe abortion even when the pregnancy would risk their life or health. Further, the clinical guidelines on contraceptive and abortion care are silent, vague, or ambiguous regarding minors’ consent. African states should remedy the situation by ensuring that clinical guidelines integrate child rights principles and standards articulated in child rights treaties to enable health providers to facilitate full, unencumbered access to contraceptive and abortion care for minor girls. A sample of clinical guidelines is analyzed to demonstrate the importance of explicit, consistent, and unambiguous language about children’s consent to ensure that healthcare workers provide sexual and reproductive health care in a manner that respects child rights.

## 1 Pregnancy and Minor Girls in Africa

Some 22years ago, this journal carried an article that expressed concern about the “widespread failure of national healthcare services to address the issue of adolescent pregnancy, childbirth and sexually transmitted infections (STIs)”^1^ (p. 13). The authors encouraged governments to comply with the international legal instruments on the rights of the child, including recognizing adolescents’ capacity to decide about their sexual and reproductive health care. Adolescent girls’ access to contraceptive and abortion care is still a pressing problem in Africa.

Adolescent pregnancy and childbearing have serious health and social consequences for girls, including complications of pregnancy and birth, child marriage, intimate-partner violence, and mental health issues.^2^ Complications in pregnancy and childbirth are the leading cause of injury and death among girls aged 15 to 19 in African countries.^3^ The risk of complications from abortion is highest in sub-Saharan Africa, where more than three quarters (77%) of all abortions are unsafe.^4^ Adolescents are more likely to experience complications than older women, because they are more likely to delay seeking an abortion, encounter barriers to accessing safer services, use less safe methods, and present late when complications arise.^5^ Yet, access to high-quality child-friendly sexual and reproductive health services provided by well-trained health providers could transform the situation.^6^ This article focuses on contraceptive and abortion care for minors.

African countries that have ratified the Protocol to the African Charter on Human and Peoples’ Rights on the Rights of Women in Africa (Maputo Protocol), the United Nations Convention on the Rights of the Child (UNCRC), and the African Charter on the Rights and Welfare of the Child (ACRWC), have an obligation to ensure that minor girls have access to contraceptive and abortion care. The legal and policy recommendations by the World Health Organization (WHO) in its 2022 abortion care guideline reinforce support for integrating child rights standards in the provision of contraceptive and abortion care.^7^

Using a sample of three countries, Malawi, Zambia, and South Africa, this article demonstrates how clinical guidelines on contraceptive and abortion care should comply with child rights standards. It comparatively analyses the drafting of the clinical guidelines and discusses how the language fosters the realization of child rights on access to contraceptive and abortion care. The discussion draws on child rights principles explained in the general comments published by three treaty-monitoring bodies: the African Commission on Human and Peoples’ Rights (ACHPR), the Committee on the Rights of the Child (CRC) and the African Committee of Experts on the Rights and Welfare of the Child (ACERWC).

In this article, ‘adolescent’ means a person between the ages of 10 and 19, while ‘child’ is a person below the age of 18, in accordance with the ACRWC^8^ (art. 2). ‘Child’ and ‘minor’ are used interchangeably. Abortion care includes health facility-based induced abortion and postabortion care.

## 2 International Legal Standards and Policies

Most African countries have ratified the UNCRC and the ACRWC. These treaties stipulate the applicable child rights standards for sexual and reproductive health care of minors. Forty-two out of the 55 countries in Africa have ratified the Maputo Protocol.^9^ The Maputo Protocol recognizes the right to sexual and reproductive health, including the rights to contraception and abortion care in cases of assault, rape, incest, and where the continued pregnancy endangers the life or mental and physical health of the pregnant girl^10^ (art. 14). Africa, therefore, has an enabling legal framework to support girls’ sexual and reproductive autonomy and their access to high-quality contraceptive and abortion care.

African governments have also adopted various regional policies to guide the realization of child rights, such as Africa’s Agenda for Children 2040, which aspires that “every child survives and has a healthy childhood”^11^ (aspiration 4). More specifically on reproductive health, the 2016–2030 Maputo Plan of Action for Implementing the Continental Policy Framework on Sexual and Reproductive Health and Rights (Maputo Plan of Action) states the following over-arching policy goal:

[t]o end preventable maternal, newborn, *child and adolescent deaths* by expanding *contraceptive use, reducing levels of unsafe abortion* (emphases supplied), ending child marriage, eradicating harmful traditional practices including female genital mutilation and eliminating all forms of violence and discrimination against women and girls and ensuring access of adolescents and youth to SRHR^12^ (para. 17).

States should bring these legal and policy instruments to bear on preventable maternal injury and deaths among minors resulting from unwanted pregnancies that are often terminated unsafely, especially in legal environments that restrict access to safe termination. States should strengthen health systems to provide quality care that aligns with the rights of the child to access contraceptive and abortion care under the UNCRC, ACRWC, and the Maputo Protocol.

The ACPHR, which monitors the implementation of the Maputo Protocol, has explained that Article 14 of the Maputo Protocol requires that states formulate precise standards and guidelines for health providers, stating that the consent of parents and their authorization is not required for the minor to access contraceptive or abortion care^13^ (para. 43). In its general comment published in 2021, the African Committee of Experts on the Rights and Welfare of the Child (ACERWC), which monitors the implementation of the ACRWC, makes similar recommendations, and explains that the age of consent should not hinder adolescents’ access to sexual and reproductive health services^14^ (para. 148). The WHO Abortion care guideline, which resonates with the ACHPR’s and the ACERWC’s recommendations, provides the rationale. The evidence shows that adolescents avoid parental authorization to escape anticipated violence, reproductive coercion and family disharmony. Further, mandated parental involvement and authorization are associated with delays in seeking care and recourse to unsafe means to terminate pregnancy^7^ (p. 43). Therefore, imposing third-party authorization and parental consent requirements when children and adolescents access safe abortion care is incompatible with child rights standards^7^ (p. 11).

## 3 Autonomy, Evolving Capacities and the Rights of the Child

Autonomy is the right of a competent adult to self-determination. In medical practice, persons exercise autonomy when they consent to or refuse care.^15^ Common law presumes adults to be capable of consenting to their health care. In contrast, in principle, children are considered incompetent and require parental consent.^16^

Protecting the child’s autonomy is the core reason the UNCRC and ACRWC were adopted^17^ (para. 11). These legal instruments recognize that children are “entitled to special protection measures and, according to their evolving capacities, they can progressively exercise their rights” to independent decision-making^18^ (Introduction). The concept of evolving capacities is central to protecting the child’s autonomy. It safeguards the independence of the child from being undermined by adult power. Evolving capacity recognizes the child’s capacity to decide about pregnancy prevention or accessing abortion care.

The CRC, the body that monitors the implementation of the UNCRC, recommends that states permit children to consent to contraception and abortion care without requiring the permission of a parent or guardian.^19^ Protecting the child’s autonomy entails that the health provider should determine whether the minor can give informed consent and then facilitate access to care for the minor, but also provide the necessary support if the minor cannot give consent.^20^

Therefore, health systems must ensure that health providers are guided and equipped to provide services in a manner that integrates child rights principles. Many African countries have enacted child protection legislation in compliance with the UNCRC and ACRWC. However, some countries have yet to incorporate fully child rights standards into policy documents and service guidelines.^21^

Through the general comments, the ACERWC and the CRC guide states on interpreting and applying provisions of the child rights treaties on thematic issues, including health. These treaty-monitoring bodies have consistently articulated four general principles on child rights that states should consider when dealing with any matter concerning children. They are elaborated here with a specific focus on contraceptive and abortion care.

### 3.1 Non-discrimination

Non-discrimination means that the minor girl should not be disadvantaged because of her status as a child. For instance, health providers should not deny minor girls access to contraceptives or abortion care. Discrimination may manifest as third-party authorization requirements disregarding the minor’s capacity to decide on her care. The CRC obligates states to ensure that girls can make autonomous and informed decisions about their reproductive health.^19^

States should also pay attention to intersectional discrimination, that is, where two or more grounds for discrimination simultaneously interact in such a way as to produce distinct and specific forms of discrimination. For instance, age, gender, and disability can intersect to produce a unique form of discrimination. Historically, girls with disabilities, especially intellectual disabilities, have been denied the right to exercise reproductive autonomy, including control over contraceptive use and pregnancy outcomes.^22^ States, therefore, should co-implement child rights treaties with other relevant treaties, including the Convention on the Rights of Persons with Disabilities (CRPD).

### 3.2 Best interests of the child

In the best interests of minors in Africa, states should take the necessary legislative, regulatory, and other measures to ensure that minors can consent to and access contraceptive and abortion care. The ACHPR and the ACERWC urge states to address high-risk pregnancies resulting from child marriages by providing access to safe abortion services.^23^ The ACERWC also recognizes the need for health services to be child and adolescent-friendly, including providing emergency contraception and safe abortion in cases of pregnancies resulting from sexual violation.^14^

### 3.3 Life, survival and development

Pregnancy, especially when unwanted, is a critical risk to the life, survival, and development of minors in Africa, compounded by the criminalization of abortion in some countries.^3^ Protecting life, survival, and development obligates states to ensure that a child can avoid a pregnancy she does not want. Access to contraception is, therefore, critical. The minor’s life should not be cut short by preventable causes such as the inability to access high-quality abortion care when pregnancy risks life or health. The ACERWC has made it clear that for pregnancies of children resulting from sexual violation, services, including termination of pregnancy, “must be regarded as an essential right in ensuring victims’ survival and development”^14^ (para. 41).

### 3.4 Participation

Participation means that children capable of forming their views can exercise the right to express freely their opinions on matters that affect them.^24^ Children should express their views on accessing and using contraception and abortion services and their experience of the quality of care.^19^ For health providers, this implies providing appropriate information to the minor in a manner that respects the minor’s maturity level and fully engages the child in decision-making according to her evolving capacities.^25^ States should ensure that clinical standards and guidelines enable health providers to deliver services that foster such participation.

## 4 Integrating Child Rights Standards and Principles in Contraceptive and Abortion Care

From the various general comments published by the ACHPR, CRC, and ACERWC, this section suggests how states could incorporate child rights in clinical guidelines on contraceptive and abortion care. These policies and guidelines should recognize evolving capacities, presume minors to be legally competent to consent to care, allow children to access care without the requirement of parental authorization, and aim to mitigate the impact of pregnancy resulting from sexual violation.

### 4.1 Recognize the evolving capacities of minors

As a general principle, states should ensure that guidelines recognize the evolving capacities of minors to make autonomous decisions about contraceptive and abortion care.^26^ Provision of care should not be based on age, but on the ability to consent. Mature children should be allowed to decide by themselves upon their care and give informed consent.

Therefore, the guidelines for health providers should include guidance on the procedures for assessing the minor’s capacity to give informed consent. An example of such guidance is the Gillick test, a tool that health providers in the United Kingdom use to assess whether the child can competently and independently decide about specific health care, such as immunization.^27^ Notably, such a tool should guide the health provider to act in the child’s best interests when the child can give informed consent or when the child cannot and needs other support.

### 4.2 Minors seeking care should be presumed legally competent

The United Nations Special Rapporteur on the right of everyone to the enjoyment of the highest attainable standard of physical and mental health, Dainius Pūras, suggested a practical application of the principle of evolving capacities. He urged states to introduce “a legal presumption of competence that an adolescent seeking preventive or time-sensitive health goods and services, including for sexual and reproductive health, has the requisite capacity to access such goods and services”^28^ (para. 60). The legal presumption of competence would secure the minor’s decision-making power based on their evolving capacities, rather than age, in health care programming.^29^

### 4.3 Parental consent should not be required for the minor to access services

The ACHPR has proposed that standards and guidelines stipulate that parental consent or involvement should not be required when minors seek contraceptive or abortion care.^13^ A practical example of the application of this standard is the determination by the South African High Court on whether it was constitutionally lawful for the Choice on Termination of Pregnancy Act, 1996 (Choice Act) to permit minors to terminate a pregnancy without parental consent or consultation.^30^ The petitioners believed that persons under 18 (children) would not have the capacity to consent to abortion services and were incapable of making such a decision in their best interests. However, in the Court’s opinion, the Choice Act permitted access to termination of pregnancy based on the capacity for informed consent rather than age. Therefore, a health provider should not limit access to termination of pregnancy for minors because they were a child. Instead, they should determine on a case-by-case basis whether the person was capable of consent. The Court thus affirmed the principle of evolving capacities inherent in the Choice Act, that a child capable of consenting could access abortion care without mandatory parental involvement.

### 4.4 Recognize the risks of pregnancy of a minor to mitigate the harms of sexual exploitation

In a joint general comment, the ACERWC and ACHPR recognized that child marriages expose girls to a high risk of pregnancy-related health complications. The two bodies enjoin states to address this challenge by providing access to safe abortion as contemplated under Article 14(2)(c) of the Maputo Protocol^23^ (para. 37).

The ACERWC also observes that many countries do not provide child and adolescent-friendly services insofar as they do not permit legal abortion, even for minors that are survivors of sexual violence.^14^ The ACERWC encourages states to ensure that sexual violence does not impact children’s opportunities to live their full potential. The treaty-monitoring body recommends that states provide emergency contraception and safe termination of pregnancies to mitigate the consequences of sexual violence and offer appropriate maternal health services and support to those who choose to continue the pregnancy^14^ (para. 145). African states should seriously consider the comments by the ACERWC and ACHPR to remove barriers to access to family planning, contraception, and safe abortion to end preventable injuries, disabilities, and maternal deaths among minor girls.

## 5 A Comparative Analysis Of Guidelines On Contraceptive And Abortion Care

This article examines and compares the contraceptive and abortion care guidelines of Malawi, Zambia, and South Africa to assess whether the guidelines are child- and adolescent-friendly. By showing the extent of compliance with child rights standards, the analysis aims to encourage African countries to consider revising their policies and guidelines to comply fully with child rights standards.

### 5.1 Contraception

Malawi’s National Policy on Sexual and Reproductive Health and Rights (SRHR Policy) states that “*Young people* shall not require parental consent for *STI services* (emphases added)”^31^ (para. 3.3.2.4). However, while the language favors the right to consent to services for young people, it limits this to STI services. It is also not precise enough about minors’ consent because it refers to young people, a category that may exclude some children with the capacity to consent.

The Standards and Guidelines for Comprehensive Abortion Care in Zambia describe consent policies for adolescents relevant to contraceptives in several statements. It requires providers to “Ensure respect of autonomy in decision-making without third party authorization”^32^ (p. 18). The standards and guidelines also state that “Providers should act in good faith in the interest of the minor and this may involve leaving out parental or guardian consent where this is not mandated by policy”^32^ (p. 19). However, the guidelines state that “mentally competent *adults* do not require the consent (authorization) of any third party (emphasis added)”^32^ (p. 8). These statements read together are ambiguous about the position of children who, by definition, are not adults. This ambiguity would be avoided if the policy stated that children with the capacity to consent do not require parental consent.

In South Africa, the National Contraception Clinical Guidelines affirm that children from the age of 12 can access contraceptive advice and services.^33^ However, specifying the age of 12 in the law could prejudice minors below the age of 12 who can give consent. This hardwiring of age in the law contradicts the principle of evolving capacities.

### 5.2 Abortion

Malawi criminalizes abortion except if it is performed to save the pregnant woman’s life. The Standards and Guidelines for Postabortion Care provide that the health provider should obtain consent from the *woman* when performing a specific procedure.^34^ Without the explicit mention of consent for minors, health providers may interpret the guidelines as excluding minors’ consent.

Zambian law permits abortion on several grounds, including risk to health and life. The Standards and Guidelines for Comprehensive Abortion Care state that a person below the legal age of consent, that is, 18years of age, requires the permission of a parent or a guardian to terminate the pregnancy. It also indicates that the child’s best interests would be considered.^32^ However, the guidelines are not explicit on whether a minor could access termination of pregnancy without the requirement of parental consent. Further, the child’s best interests principle is open to various interpretations, meaning that health providers are left to do the interpreting without concise guidance.

South African abortion law permits abortion on request up to 12weeks of gestation. South Africa’s National Clinical Guideline for Implementation of the Choice on Termination of Pregnancy Act stipulates that there is no minimum age of consent for access to safe abortion.^35^ It further states that there is no requirement for third-party authorization for a girl who can competently consent to an abortion. Such framing is in line with the recommendations in the WHO abortion care guideline on providing high-quality abortion care to minors. It integrates the principle of evolving capacities envisaged in the UNCRC and the ACRWC, and enables mature children to consent to abortion care without mandatory parental involvement.

### 5.3 Enhancing the compliance of guidelines with child rights standards

Unlike other healthcare services, contraception, abortion, and post-abortion care are stigmatized, moralized, and influenced by social norms about sexuality, particularly when serving children.^36^ The absence of explicit language about minors’ consent may prejudice minors because if there is uncertainty, “Healthcare providers end up using personal discretion on ‘an appropriate age’ instead of practising within the legal framework”^37^ (p. 10). Therefore, to facilitate compliance with child rights standards, clinical guidelines should stipulate consent for minors with precision to leave no room for ambiguity.

Laws and policies may use general and broad language such as ‘all sexually active persons’ have access to ‘any’ contraceptive method of their choosing or state general principles such as that the health providers shall consider ‘the best interests of the child’. However, guidelines would be most helpful for health providers, who are the front-line workers, if they used precise language regarding minors’ consent to contraceptive and abortion care and the nonrequirement of third-party authorization. In cases where the child cannot consent to care, the guidelines should not leave things in abeyance but stipulate how the health provider would facilitate access to care for the minor in that situation, even if the minor does not want parental involvement.

Therefore, Malawi and Zambia should consider revising and improving their contraceptive and abortion care guidelines to introduce concise language on minors’ consent. The guidelines must also explicitly indicate that parental consent is not mandatory for children who can consent. Meanwhile, states could train health providers to apply child rights standards in adolescent sexual and reproductive health care.

While South Africa has such precise language in guidelines on abortion care, the law on contraceptives has set the age limit of 12. South Africa should consider that the law prejudices competent children below the age of 12 and remove the minimum age.

## 6 Support for Health Providers

Health providers are vital to the delivery of quality contraceptive and abortion care. States should support health providers by ensuring that the language of consent for minors in policies and clinical guidelines is consistent with child rights standards.

States should also clarify the application of criminal laws that impact access to contraception and safe abortion for minors. For example, countries that legally restrict access to termination of pregnancy may make an exception for children to mitigate the impact of pregnancies resulting from sexual exploitation, as recommended by the ACERWC. However, suppose states do not clearly explain the exception to health providers. In that case, the criminal law may have a chilling effect on health providers and interfere with their duty to provide care to minors eligible for legal abortion.

Beyond ensuring that the language of clinical guidelines is consistent with child rights, states should explore effective ways to address bias and discrimination in provision of contraceptive and abortion care to minors.^38^ The approaches to improving care should be supportive of health providers rather than encouraging blaming them; for instance, states should avoid using penal sanctions as a means of compelling health providers to comply with human rights standards^39^ (sec. 20(2)).

## 7 Conclusion

African states should implement measures to ensure that contraceptive and abortion care comply with child rights treaties and the Maputo Protocol to eliminate preventable maternal deaths among African children and adolescents. African states should create enabling legal and policy frameworks, including drafting concise clinical guidelines, and implement plans to support health providers in delivering contraceptive and abortion care to children who need these services in a manner consistent with child rights.
